# Unveiling the Uncommon: Superior Mesenteric Artery Syndrome Presenting As Gastritis

**DOI:** 10.7759/cureus.40704

**Published:** 2023-06-20

**Authors:** Noman Salih, Khalid S Baig, Izhar Ullah, Numan Ghani, Yamna Ali, Shahid Ali, Shehzad A Khan, Muhammad Ihtisham

**Affiliations:** 1 Internal Medicine, Hayatabad Medical Complex, Peshawar, PAK; 2 Internal Medicine, Khyber Teaching Hospital, Peshawar, PAK; 3 Internal Medicine, Lady Reading Hospital, Peshawar, PAK; 4 Internal Medicine, Khyber Medical Center, Peshawar, PAK

**Keywords:** esophago-gastro-duodenoscopy, tuberculosis, gastritis, hematemesis, superior mesenteric artery syndrome

## Abstract

Superior mesenteric artery syndrome (SMAS) is a specific type of duodenal obstruction marked by a blockage of the inferior part of the duodenum as a result of compression between the superior mesenteric artery (SMA) and the aorta. Depletion of the mesenteric fatty pad causes this complication. In the current study, we describe a case of SMAS involving a 36-year-old lady who presented with postprandial pain, weight loss, and hematemesis. The patient was investigated for chronic pancreatitis, celiac disease, and intestinal tuberculosis due to a vague presentation, which yielded normal results. Subsequently, esophagogastroduodenostomy (EGD) was performed during a follow-up visit, which revealed erosive gastritis and antral inflammation. The patient was eventually given the go-ahead for a CT scan which led to the diagnosis of SMAS leading to erosive gastritis and distal duodenal obstruction.

## Introduction

The first description of superior mesenteric artery syndrome (SMAS) was made by Carl von Rokitansky in 1861. David Wilkie then described the condition's precise clinical and pathological features and gave it the name chronic duodenal ileus [1]. SMAS is an upper GI tract complication caused by potentially fatal duodenal compression. It often manifests as a quick and significant reduction in weight, with the most noticeable complaints being feeling full sooner, postprandial pain, as well as bile retention [2]. Rarely, SMAS can present as hematemesis secondary to erosive gastritis, as observed in our case, making it unique in comparison to other available cases in the literature. In most cases, making a diagnosis is difficult because the symptoms are present in multiple other conditions such as intestinal tuberculosis(TB), celiac disease, and chronic pancreatitis. The best method for diagnosing SMAS is debatable because the complaints are frequently irrelevant or inconsistent with the diagnostic radiology findings. Additionally, following treatment in many instances, the complaints do not completely disappear [1]. As a result, the issue is typically not properly recognized or is mistakenly linked to other structural or motility-related reasons for duodenal obstruction [3]. Infectious diseases, such as intestinal TB and Chagas disease, also need to be ruled out.

## Case presentation

In this study, we discussed a 36-year-old female patient who presented to our emergency department with the chief complaint of blood in the vomitus. This was her third episode in the last 24 hours. Previously, she visited us about three months ago and complained of severe epigastric discomfort, nausea, indigestion, and weight loss (approximately five kgs) for the last five months. She had colicky epigastric pain that wasn't distributed to other parts of her abdomen and did not exhibit any symptoms of difficulty swallowing. Furthermore, she did not have any previous history of cancer, pulmonary tuberculosis, burns, absorption problems, spinal cord trauma, neurological problems, or prolonged hospitalization.

Apart from an emaciated look, there were no other positive findings on the examination. Her baseline investigations shown in Table 1 were normal. Ultrasound abdomen and pelvis was done which did not reveal any pathology. She was investigated for celiac disease, chronic pancreatitis, and intestinal TB due to nausea, weight loss, and indigestion; however, these were ruled out after negative anti-tissue transglutaminase IgA and IgG for Celiac disease, normal amylase and lipase for chronic pancreatitis later confirmed by CT, and stool Gene Xpert as well as normal ultrasound for intestinal tuberculosis, which was also excluded later by CT of the abdomen and pelvis. She was sent home with the advice of returning to the hospital for a contrast-enhanced CT of the abdomen and pelvis because of the unavailability of contrast medium in the hospital for which she had to wait. A few days later, she presented with three episodes of mild hematemesis and slight dehydration, for which she was prophylactically administered vitamin K and intravenous Transamin, intravenous fluids, and antiemetics, which relieved her symptoms. Baseline investigations along with a coagulation profile were repeated as shown in Table 1 in the follow-up column.

**Table 1:** Hematologic Investigations on admission and follow-up ABBREVIATIONS: WBC, white blood cell; Hb, hemoglobin; PLT, platelets; Na, sodium; K, potassium; Cl, chloride; PT, prothrombin time; APTT, activated partial thromboplastin time; INR, international normalized ratio; ALT, alanine aminotransferase; ALP, alkaline phosphatase.

Test	Reference	Initial presentation	Follow-up
WBC (/µl)	4000 -11000	12200	12800
Hb (gr/dl)	11.5 - 17.5	12.6	12.1
PLT (/µl)	150000 - 450000	317000	305000
Na (mmol/l)	135 - 150	139.9	141.5
K (mmol/l)	3.5 -5.1	4.5	3.9
Cl (mmol/l)	96 - 112	109.5	102.6
PT (sec)	12	-	12
APTT (sec)	28	-	28
INR	1	-	1
Amylase (IU/L)	<90	35	-
Lipase (IU/L)	13 - 60	18	-
Bilirubin (mg/dl)	0.1 - 1	0.5	0.3
ALT (IU/L)	10 - 50	12	16
ALP (IU/L)	<390	65	54

**Figure 1:**
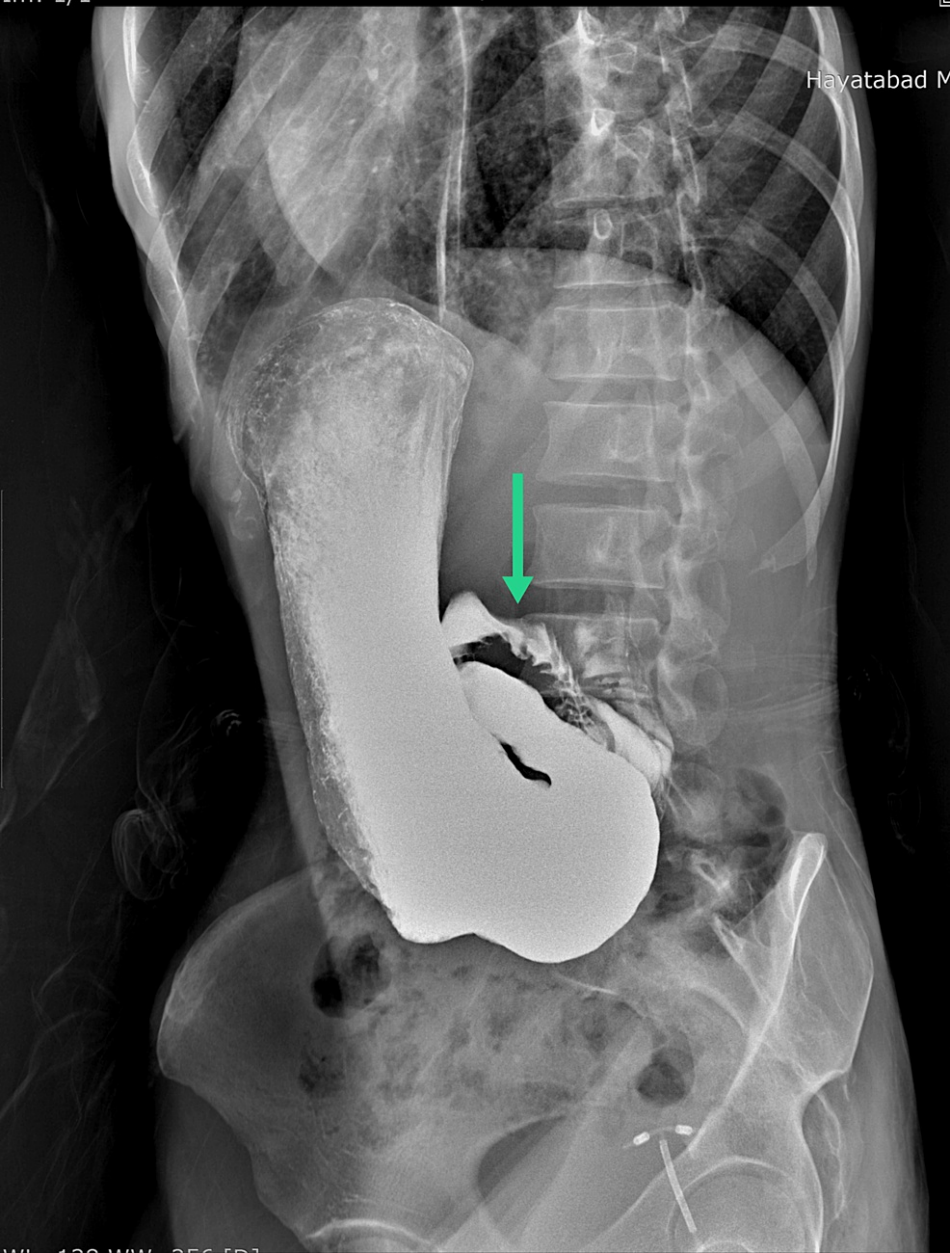
Opacification till the first part of the duodenum with no spillage of dye distally(green arrow)

A routine examination(R/E) of her stool to rule out any occult blood showed red blood cells (RBCs) in it. A repeat abdominal ultrasound performed along with an X-ray of the erect abdomen did not reveal any positive findings as there were a lot of gut gases masking the visualization of internal organs. Antral erythema and erosive gastritis were observed after the patient underwent EGD for hematemesis. Although invasive, we performed EGD before CT because of the unavailability of contrast medium for the CT. The patient was readmitted to the hospital for further work-up. During this admission, we managed to arrange for contrast for barium meal and follow-through, as well as CT of the abdomen and pelvis. Barium meal and follow-through showed opacification till the first part of duodenum with no outlining distally (Figure 1).

**Figure 2:**
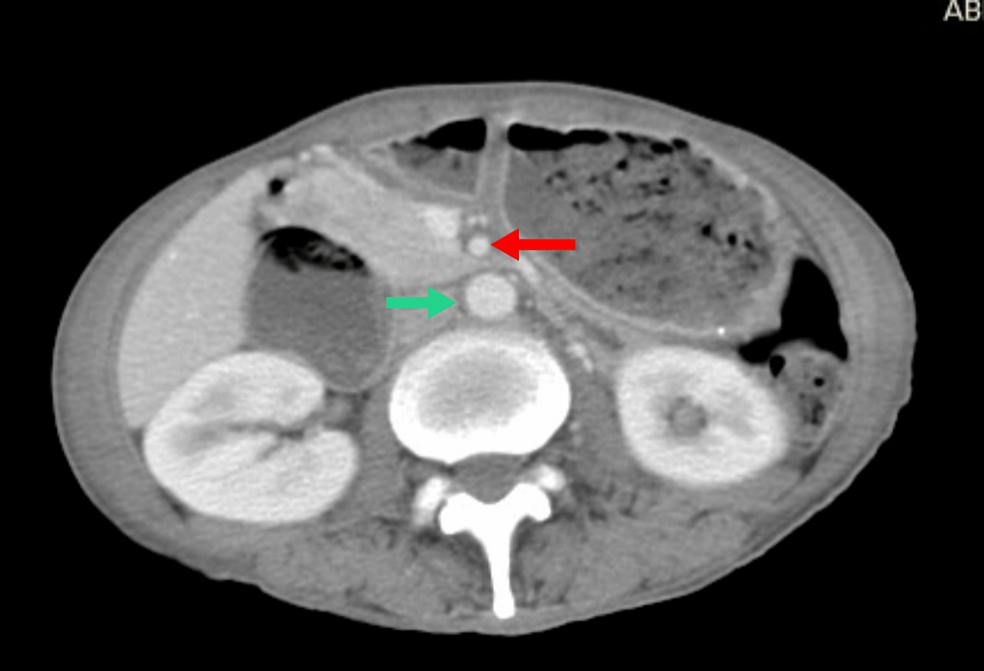
Part of the duodenum compressed between SMA (red arrow) and aorta (green arrow)

To rule out structural abnormalities, contrast-enhanced computed tomography (CT) of the abdomen and pelvis was performed. CT showed that the first section of the duodenum was expanded, with the angle between the SMA and aorta being around 20°, and the aorto-mesenteric distance measuring between 5-7 mm, according to the CT findings (Figure 2).

A colonoscopy was performed to rule out other causes of the red blood cells in the stool but the results were normal. The patient was scheduled for evaluation by the surgery department to confirm the diagnosis of SMAS as well as manage her accordingly. The patient was subsequently transported to the surgery ward where laparoscopy was performed. A review by the surgical team confirmed an extreme drop in the angle between her aorta and SMA and compression of the distal duodenum with dilation of the proximal duodenum. The jejunum, however, appeared to be healthy. The distal duodenum was compressed, and the SMA was prominent and lying over it. Duodenojejunostomy was performed and an anastomosis from side to side was created. A methylene blue test revealed no leakage. The patient recovered remarkably well postoperatively. She was administered IV antibiotics, IV fluids, and IV antacids to speed the recovery, and was able to start drinking and consume a soft diet on the fifth day of the operation. A follow-up visit two weeks later showed improvement in oral intake and symptoms, and that she was doing well. On her subsequent follow-up visits, her condition remained stable and she did not report any new symptoms.

## Discussion

The third section of the duodenum is found only in the root of the mesentery, behind the SMA, and in the front of the abdominal aorta. The duodenum is located in the space between both these vessels with a diameter of 10-28 mm, and the angle that separates the SMA and aorta is approximately 25-60°. In our case, the cavity diameter in the SMAS was reduced to 5.7 mm and the angle was decreased to 20°. As a result, the descending duodenum appeared compressed and dilated in the CT images of the aorta and SMA. A short Treitz ligament, the ectopic origin of the SMA, superior and aberrant positioning of the Treitz ligament, and reduction in the retroperitoneal fat, which typically cushions the SMA and aorta, are additional prevalent reasons for the SMAS [1, 4, 5]. More than 400 instances of superior mesenteric artery syndrome have been recorded thus far [2], with incidence higher in women than men [3]. Studies have shown that symptoms typically appear during the acute stage of the illness, which can be seen in 0.130%-0.31% of upper GI barium studies [1]. 

SMAS has also been noted in diseases such as reconstruction surgery for scoliosis [6], celiac axis compression syndrome [7], and nutcracker syndrome [8] that cause modest weight loss. SMA syndrome can also be brought on by trauma, burns [5, 9], weight loss surgery [10], injuries to the spinal cord, paraplegia [11], using abusive substances [12], and even anorexia nervosa [13]; however, these disorders result in only mild weight loss.

Both the main cause and level of obstruction influence symptom severity. Primarily, postprandial pain along with early fullness may be observed in moderate cases; however, patients having severe SMAS may experience weight loss and hemoptysis, as in our case, which occurred as a result of gastritis secondary to SMA syndrome. The lateral decubitus and prone positions relieve pressure on the mesentery and SMA and widen the gap between the aorta and the SMA. It is generally accepted that symptoms may disappear when a patient is lying in the left and right lateral or prone positions. However, our patient did not report this occurrence [1]. Abdominal distention, succussion splash, with increased bowel noises, are among the nonspecific physical examination findings. Individuals who vomit or regurgitate a lot may experience serious electrolyte imbalances, like metabolic alkalosis [14]. Delay in diagnosis may cause consequences, including intestinal perforation, portal venous gas, gastrointestinal pneumatosis, bezoar formation due to duodenal blockage, electrolyte imbalances, or mortality [14, 15]. A few instances of conservative treatment include the insertion of a nasogastric tube to lower pressure in the duodenum and the management of imbalances in electrolyte levels like metabolic alkalosis. Thus, in order to help patients with severe blockage to gain weight, nasojejunal tubes for feeding may be necessary [16]. When nutritional support is added to conservative treatment, it often works well for older adults with mild illnesses and in children whose conditions are likely to be acute. However, for individuals with chronic diseases, dietary support is insufficient. Patients with SMA syndrome should have their electrolyte abnormalities corrected and get nutrition briefly through a nasogastric tube. Numerous surgical procedures, such as duodenojejunostomy, gastrojejunostomy, as well as Strong's surgery, in which the ligament of Treitz is sectioned to release the compressed part of the duodenum from the space between the aorta and mesentery, may be taken into consideration if the symptoms do not go away [9, 16]. Duodenojejunostomy is the most popular surgical procedure, and it is carried out using transabdominal or laparoscopic techniques; however, the laparoscopic approach is used during duodenojejunostomy because of its high likelihood of effectiveness and minimal likelihood of problems, such as ulcers [3, 17].

## Conclusions

We encountered an instance of SMA syndrome leading to gastritis, which presented as hematemesis leading to difficulty in diagnosis. Substantial duodenal enlargement related to SMA syndrome results in vomiting, malabsorption, and weight loss. Ischemia of the gastric wall secondary to duodenal and gastric distention is the main cause of gastritis which led to hematemesis. Initially, we considered chronic pancreatitis, celiac disease, and intestinal TB, which were ruled out. Contrast-enhanced CT of the abdomen and pelvis was done to rule out any structural disease that led to the confirmation of SMA syndrome. Although conservative therapy can help in less severe cases, we opted for surgery to repair the duodenal compression because of the severity of our case. By looking at our case, physicians will consider SMAS early in their differentials while doing workups for abdominal pain and weight loss, and will hopefully prevent it from progressing further leading to gastritis.
